# CrackNet: A Hybrid Model for Crack Segmentation with Dynamic Loss Function

**DOI:** 10.3390/s24227134

**Published:** 2024-11-06

**Authors:** Yawen Fan, Zhengkai Hu, Qinxin Li, Yang Sun, Jianxin Chen, Quan Zhou

**Affiliations:** 1 National Engineering Research Center of Communications and Networking, Nanjing University of Posts & Telecommunications, Nanjing 210003, China; 2 Artificial Intelligence of Key Laboratory of Sichuan Province, Sichuan University of Science and Engineering, Yibin 644000, China; 3 College of Harbour, Coastal and Offshore Engineering, Hohai University, Nanjing 210098, China

**Keywords:** crack segmentation, hybrid model, class imbalance, dynamic weight loss

## Abstract

Cracks are a common form of damage in infrastructure, posing significant risks to both personal safety and property. Along with the development of deep learning, visual-based crack automatic detection has been widely studied. However, this task is still challenging due to complex crack topology, noisy backgrounds, unbalanced categories, etc. To address these challenges, this research proposes a novel hybrid network, named CrackNet, which leverages the strengths of both CNN and transformer. On the encoder side, CNNs are employed to extract multi-level local features, while transformers are used to model global dependencies. Additionally, a strip pooling module is introduced to suppress irrelevant regions and enhance the network’s ability to segment narrow and elongated cracks. On the decoder side, an attention-based skip connection strategy and a mixed up-sampling module are implemented to restore detailed information. Furthermore, a joint learning loss combining Dice and cross-entropy with dynamic weighting is proposed to mitigate the effects of severe class imbalance. CrackNet is trained and evaluated on three public crack datasets, and experimental results show that the proposed model outperforms several well-known deep neural networks, with a particularly noticeable improvement in recall rate.

## 1. Introduction

Crack detection has become increasingly critical in recent years due to heightened concerns regarding infrastructure safety and maintenance. During both construction and long-term operation, the formation of cracks is often accelerated by material deterioration and environmental factors such as temperature fluctuations and heavy traffic. If these surface defects remain undetected, they can compromise structural integrity and pose significant safety risks [1]. Traditional manual inspection methods typically involve sketching and documenting cracks by hand. However, this approach is labor-intensive, time-consuming, and subject to environmental influences, making it challenging to ensure the reliability of results. Additionally, certain areas, such as the undersides of bridges, are difficult to access through manual inspection. Recent advancements in computer vision and machine learning, present effective solutions for automating crack detection [2]. These technologies enable timely and accurate evaluations, thereby enhancing maintenance practices and extending the lifespan of critical infrastructure.

**Figure 1:**
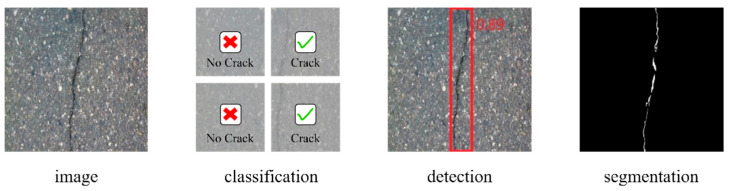
Crack Detection Tasks.

Given the importance and complexity of this problem, extensive research has been conducted over the past few decades. Recently, deep learning (DL)-based crack detection methods have demonstrated superior performance [3,4,5,6,7] and can be broadly classified into three categories [2] within the field of computer vision, as shown in Figure 1. The crack classification model needs to determine whether an image contains a crack or not. Despite being an image-level binary classification task, it is still challenging due to the presence of other surface defects that may appear similar to cracks. The purpose of crack detection is achieved using bounding boxes to automatically identify the presence and location of cracks in images. There are usually two types of methods for crack detection: one is the two-stage method represented by Fast R-CNN [8], and the other is the one-stage method represented by YOLO [9]. Crack segmentation refers to the process of separating and extracting pixel-wise crack regions from other parts in an image, which can be used to obtain the quantification of parameters, such as crack length and width of cracks. Therefore, crack segmentation models are preferred for their ability to provide the most valuable information for crack risk level assessment. These advantages make crack segmentation the current deep learning trend in automated crack detection.

However, crack segmentation is an extremely challenging problem. Firstly, cracks are widely distributed on the surfaces of various components of civil infrastructure, such as bridges, dams, and roads, which can lead to issues like intensity inhomogeneity, low contrast, and cluttered backgrounds in crack images. Additionally, cracks are typically elongated and narrow, yet topologically complex. This not only makes it difficult to segment fine cracks but also results in class imbalance, causing the model to be biased toward segmenting the background. This bias is reflected in segmentation results that have lower recall rates. Moreover, the widespread distribution of cracks and many inaccessible scenes make it challenging to collect sufficient crack image data. While unmanned aerial vehicles have partially alleviated this issue, there remains a need to maximize the use of the currently limited dataset for groundbreaking work.

Motivated by the discussions above, we propose a novel model named CrackNet for crack segmentation. This model combines convolutional neural networks and transformer blocks. The convolutional module helps to extract local detail features of the cracks, aiding in the segmentation of fine cracks, while the transformer leverages long-range dependencies to address the complex topological structures of the cracks. The proposed method consists of three modules: Encoder Module (EM), Decoder Module (DM), and Attention Module (AM). Specifically, to deal with the class imbalance issue, a dynamic weight loss is introduced.

The main contributions of this paper can be summarized as follows:

1. CrackNet proposes a novel architecture in which: (1) The encoder structure is based on an improved hybrid model of CNN and transformer, enabling the establishment of remote dependencies at a high level while retaining local feature extraction capabilities; (2) A novel spatial attention module is added to each skip connection. This module emphasizes the overlapping regions of two feature maps and supplements fine-grained features, effectively reducing background interference; (3) We propose using strip pooling instead of traditional square pooling. This adaptation is specifically tailored to the elongated nature of cracks, enhancing the model’s ability to detect fine, narrow features while minimizing interference from irrelevant background noise.
2. We introduce a dynamic loss function to address the issue of imbalanced data distribution, in which the loss weights are dynamically updated based on the model’s different learning stages. This adaptive approach not only enhances the model’s performance by improving recall rates for underrepresented classes, but also reduces the likelihood of overfitting to the dominant classes. Furthermore, the proposed weight updating mechanism can be easily extended to other application scenarios, making it a versatile solution for various tasks involving imbalanced datasets.
3. The proposed method has been validated on multiple public crack datasets, demonstrating overall performance superior to that of other models.

The rest of this paper is organized as follows. In Section 2, we review research related to the proposed method. In Section 3, we provide an overview of the proposed network structure and loss function for refined crack detection. Section 4 presents implementation details, evaluation metrics, experimental results, and visualization analysis. Section 5 concludes the paper.

## 2. Related Work

In this section, we briefly review recent methods for crack segmentation, broadly categorized into CNN-based, transformer-based, and hybrid models. We also review some recent methods to deal with class imbalance.

### 2.1. CNN-Based Segmentation Network

In recent years, there have been numerous CNN-based methods applied to crack segmentation, such as CrackU-net [10], DeepCrack [11], FPHBN [12], and SCHNet [13]. In particular, SegNet [14], U-Net [15], and their variants [16,17,18,19], which adopt an encoder–decoder structure [20,21], have achieved significant performance. Liu et al. [11] proposed a deep hierarchical feature learning architecture for crack segmentation, with multiple convolutional neural network layers and a multi-level feature fusion module. Based on the faster-RCNN model, Kang et al. [22] employ different bounding boxes and a modified tubularity flow field (TuFF) algorithm to segment crack pixels from detected regions and measure the crack thickness and length. Chu et al. [23] proposed a multi-scale feature fusion network named Tiny-Crack-Net that utilizes attention mechanisms for the segmentation of tiny cracks.

Although CNN-based methods have achieved competitive performance in crack segmentation, the limited receptive field of the convolution operation causes the loss of global information during feature extraction. This dramatically reduces the feature expression of tiny cracks in the down-sampling process and leads to discontinuities and incorrect details in the detection of long and thin cracks.

### 2.2. Transformer-Based Segmentation Network

Compared to CNN-based methods, transformers have demonstrated strong global modeling ability through a multi-head self-attention mechanism [24] and have been successfully applied in computer vision [25]. Specifically, Vision Transformers (ViTs) [25] have performed well in various tasks such as image classification, object detection, and semantic segmentation, with examples including Swin Transformer [26] and Reformer [27]. ViT-based frameworks have also been applied to crack segmentation [28,29,30]. Qi et al. [31] developed an end-to-end model that combines Vision Transformer (ViT) with level set theory for segmenting defects in bridge pavements. The model achieves precise segmentation by integrating the outputs of two parallel decoders.

However, despite various optimization schemes proposed by researchers to reduce transformer computations, it still requires pre-training on large-scale datasets, which is extremely labor-intensive and resource-consuming. Additionally, transformers lack specialized mechanisms for processing local information, which plays a significant role in crack segmentation tasks.

### 2.3. CNN and Transformer Hybrid Segmentation Network

To address these challenges, a hybrid model combining Convolutional Neural Networks (CNNs) and transformers may offer an effective solution. However, determining how to best integrate these two feature types remains an important question. Chen et al. [32] introduced TransUNet, a pioneering network architecture that merges the strengths of both transformers and CNNs for medical image segmentation. In TransUNet, only simple skip connections are employed without incorporating attention mechanisms, which hinders feature fusion and detail recovery. This is particularly critical for the segmentation of fine cracks. Similarly, Swin-Unet [33] also adopts a U-Net-inspired structure, but it specifically utilizes the Swin Transformer to enhance the model’s ability to efficiently capture both local and global features. However, the Swin-Unet algorithm necessitates a substantial amount of data to achieve optimal performance. When data availability is limited, it falls short compared to convolutional networks.

Unlike the above implicit fusion of the two feature types, UCTNet [34] leverages the stability of anatomical structures in medical images by explicitly assigning transformers to focus solely on establishing global dependencies in the CNN’s uncertain regions. This approach minimizes functional overlap between the CNN and transformer, enhancing overall performance. The effectiveness of hybrid models has also been demonstrated in the field of crack segmentation [35,36,37]. Particularly, Wang et al. [38] proposed a dual-path segmentation network, named Crackmer, that combines CNN and transformer for crack segmentation. The network is designed with a pyramid structure, enabling hierarchical extraction of features at different scales.

Thus, the objective of this research is to explore a compact hybrid model to develop a network that improves crack segmentation accuracy while maintaining a lightweight architecture.

### 2.4. Loss Function

In crack segmentation tasks, class imbalance is a common and critical challenge. Since cracks typically occupy only a small portion of the image while the background takes up the majority of the area, class imbalance often arises during training, which negatively impacts model performance. To address this issue, many studies have proposed various methods from the perspective of loss functions to mitigate the effects of class imbalance.

Weighted cross-entropy (WCE) as a straightforward tool has been used to solve this class imbalance problem, especially in the end-to-end model for segmentation. For instance, Xie et al. [39] employed the ratio of ground-truth edge and non-edge samples in a training batch to reweight the non-edge and edge samples in the weighted cross-entropy (WCE) loss. Similarly, Fang et al. [40] utilized an adaptive WCE approach to address category imbalance. Li et al. [41] proposed three novel adaptive WCE losses for dealing with crack detection. In crack segmentation tasks, to further enhance model performance, researchers have proposed hybrid loss functions that combine the advantages of different loss functions. For instance, a combination of cross-entropy loss and Dice loss can address both class imbalance and the similarity of the segmented regions, thereby improving the model’s robustness [38]. But in these studies, the weights were fixed once they had been learned and did not take into account the different stages of learning.

## 3. Methods

### 3.1. Overview of the Proposed Method

**Figure 2:**
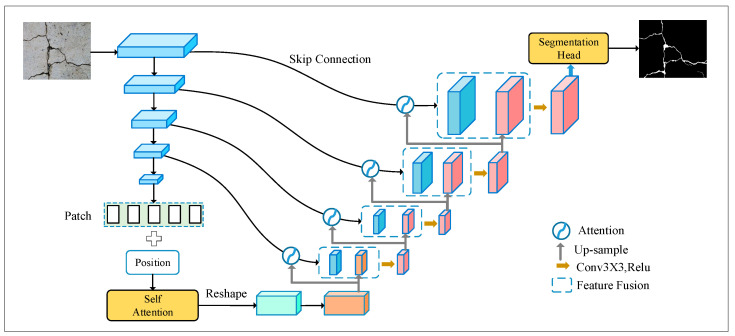
Overview of the proposed CrackNet.

In this paper, CrackNet is proposed for crack segmentation, which consists of an encoder and a decoder (outlined in Figure 2). The encoder mainly includes CNN module, transformer module, and strip pooling module. CNN module is used to extract multi-scale feature maps. The transformer module is used to explore global contextual information to help the model locate cracks in complex backgrounds, while the strip pooling module is primarily designed to address the problem of background interference caused by unrelated cracks in traditional square-kernel convolutions. The decoder is mainly composed of multiple cascaded up-sampling modules. Specifically, this paper uses a hybrid up-sampling method (combining nearest up-sampling and bilinear interpolation up-sampling), and adopts attention modules to better utilize the information carried by the skip connections.

### 3.2. Hybrid Encoder

In the encoder end, a hybrid model is adopted that integrates convolutional and transformer mechanisms to effectively capture the long-range interactions across different segments of a crack image, leveraging both local and global insights. This methodology comprises three key components: CNN-Module, Trans-Module, and strip pooling. These elements are strategically employed to investigate and understand the intricate connections among diverse portions of the crack.

(1) CNN-Module

As shown in Figure 2, the CNN module consists of several convolutional layers, which generate feature maps at different resolutions. Each layer is composed of two consecutive convolutional blocks followed by a max-pooling layer with a step size of 2 to generate next resolution feature map. The process of the CNN-module can be formulated as follows:

(1) $$\left\{\begin{array}{l} F_{e}^{1}=\mathrm{ConvBlock}(X)\\ F_{e}^{l}=\mathrm{ConvBlock}(\mathrm{MaxPool}(F_{e}^{l-1})),\;l=2,\cdots L-1\\ F_{e}^{L}=\mathrm{ConvBlock}(F_{e}^{L-1}) \end{array}\right.\;,$$

where X represents the input image, and $F_{e}^{l}$ denotes the feature map of the l−th layer. The L−th feature is subsequently input into the Trans-Module, and other layer’s feature maps are linked to decoder through skip connections.

(2) Trans-Module

**Figure 3:**
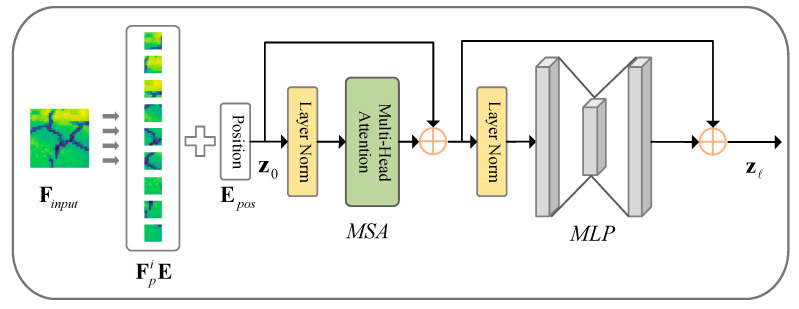
Transformer Module.

The architecture of the Trans-Module is depicted in Figure 3. Here, we take the convolutional feature map at its lowest resolution as the input, denoted as $F_{input}\in {\mathbb{R}}^{H\times W\times C}$, where H×W specifies the dimensions of the input feature map, and *C* indicates the number of channels. The process begins by dividing the input into *N* patches of size P×P, and subsequently reshaping each patch into a 1-dimensional vector. These patches are then flattened $\left\{F_{p}^{i}\in {\mathbb{R}}^{P^{2}\cdot C}|i=1,\cdot \cdot \cdot ,N\right\}$ and subjected to a linear transformation to project them into *D*-dimensional space. To preserve the spatial information of these patches, this article selects a learnable position encoding to generate the final visual tokens, as described below:

(2) $$z_{0}=\left[F_{p}^{1}E;F_{p}^{2}E;\cdot \cdot \cdot ;F_{p}^{N}E\right]+E_{pos},$$

where $E\in {\mathbb{R}}^{\left(P^{2}\cdot C\right)\times D}$ represents the trainable linear mapping, $E_{pos}={\mathbb{R}}^{N\times D}$ denotes the position embedding. $z_{0}$ serves as the input to the transformer block, which is composed of *L* blocks of multi-head attention (MSA), and multi-layer perceptron (MLP). The process of transformation can be expressed as follows:

(3) $$z_{l}^{\prime }=\mathrm{MSA}(\mathrm{LN}(z_{l-1}))+z_{l-1},\;l=1\cdot \cdot \cdot L$$

(4) $$z_{l}=\mathrm{MLP}(\mathrm{LN}(z_{l}^{\prime }))+z_{l}^{\prime },\;l=1\cdot \cdot \cdot L$$

where LN denotes the layer normalization operator.

### 3.3. Bottleneck Layer

Cracks are typically characterized by their narrow features. However, when utilizing standard pooling methods, there is an inevitable inclusion of numerous irrelevant areas, especially when dealing with objects of irregular shape. This makes it obvious that standard pooling techniques fall short in effectively processing crack patterns. Consequently, strip pooling [42] is introduced to extract features specifically from the horizontal and vertical dimensions.

**Figure 4:**
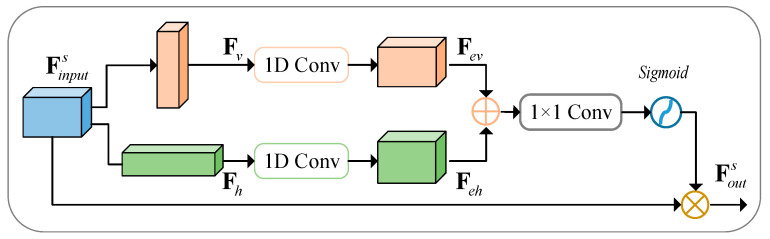
Strip pooling module.

The strip pooling strategy, as depicted in Figure 4, involves several key steps. First, the output feature of the transformer block, denoted as $z_{l}$, is reshaped into the same dimension as $F_{e}^{L}$. Next, it is compressed along the horizontal and vertical directions using average pooling, resulting in two new feature maps $F_{v}\in {\mathbb{R}}^{H\times 1\times C}$ and $F_{h}\in {\mathbb{R}}^{1\times W\times C}$. Following this, 1D convolution with a kernel size of 3 is applied to expand the pooled maps back to their original size, resulting in the expanded feature maps $F_{ev}\in {\mathbb{R}}^{H\times W\times C}$ and $F_{eh}\in {\mathbb{R}}^{H\times W\times C}$. These expanded feature maps are then summed pixel-wise. Finally, after undergoing 1 × 1 convolution and sigmoid processing, the resulting feature map is multiplied with the original input feature map at corresponding pixels to obtain the ultimate output result $F_{out}^{s}\in {\mathbb{R}}^{H\times W\times C}$. The entire process can be formulated as follows:

(5) $$F_{out}^{s}=\sigma \left({\mathrm{Conv}}^{1\times 1}\left(F_{ex}⊕F_{eh}\right)\right)⊗F_{input}^{s},$$

where $\sigma \left(\cdot \right)$ refers to sigmoid function, ⊕ denotes element-wise add, and ⊗ is element-wise multiplication.

### 3.4. Attention Decoder Module

The attention-driven decoder plays a crucial role in reconstructing the crack region and is composed of the mixed up-sampling block, attention block, and convolutional block. At each stage of the decoder, a sequential operation involving mixed up-sampling and convolution is applied. To effectively leverage the encoder features obtained via skip connections, an attention module is integrated into the decoder architecture. This comprehensive design ensures that the reconstruction process is guided by attention mechanisms that enhance the utilization of relevant information from the encoder, ultimately contributing to the accurate reconstruction of the crack region.

Given the output feature of the decoder $F_{d}^{l-1}\;\in \;{\mathbb{R}}^{H/2\times W/2\times C}$, it undergoes parallel up-sampling using linear and nearest neighbor methods, resulting in ${\hat{F}}_{l-up}^{l}\in {\mathbb{R}}^{H\times W\times C}$ and ${\overset{⌢}{F}}_{n-up}^{l}\in {\mathbb{R}}^{H\times W\times C}$, respectively. These two feature maps are then merged to obtain the final up-sampling feature map $F_{up}^{l}={\hat{F}}_{l-up}^{l}⊕{\hat{F}}_{n-up}^{l}$.

**Figure 5:**
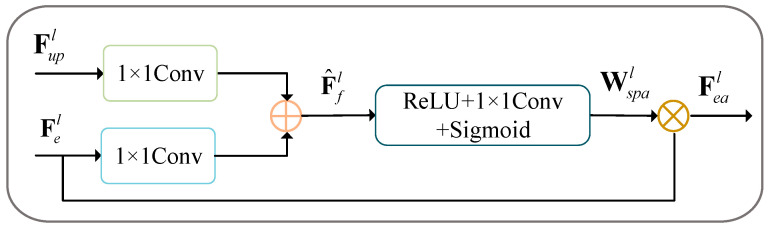
Attention block.

To achieve a more precise segmentation of cracks, a novel spatial attention module (Figure 5) is proposed and appended to each skip connection, which emphasizes the overlapping regions of two feature maps but also supplements fine-grained features, effectively reducing background interference. Given the encoder feature $F_{e}^{l}\in {\mathbb{R}}^{H\times W\times C}$ and the up-sampling feature $F_{up}^{l}\in {\mathbb{R}}^{H\times W\times C}$, they are first passed through a 1×1 convolution layer, with channel dimension reduced from H×W×C to H×W×C/2. And then an element-wise addition is applied to generate the fused feature map ${\hat{F}}_{f}^{l}\in {\mathbb{R}}^{H\times W\times C/2}$. Subsequently, the attention feature map $W_{spa}\in {\mathbb{R}}^{H\times W}$ is activated by ReLu, followed by a 1 × 1 convolution layer and Sigmod activation.

(6) $$W_{spa}=\mathrm{Sigmod}\left({\mathrm{Conv}}^{1\times 1}\left(\mathrm{ReLu}(\hat{F})\right)\right).$$

The output of the attention module is calculated as follows:

(7) $$F_{ea}^{l}=W_{spa}^{l}⊗F_{e}^{l}.$$

Furthermore, the *l*th layer feature map of the decoder is computed as follows:

(8) $$F_{d}^{l}=\mathrm{ConBlock}(\mathrm{Cocat}(F_{ea}^{l},F_{up}^{l})).$$

Finally, the feature map passed through a 1×1 convolution layer and a sigmoid layer to obtain the segmentation results.

### 3.5. Dynamic Loss Function

The task of crack detection differs from others due to its small and elongated characteristics. This causes an issue of extremely unbalanced categories in the image.

Hence, a mixed loss function that combines adaptive weighted cross-entropy (WCE) [37] and Dice [38] is proposed to alleviate this issue,

(9) $$L=L_{Dice}+L_{wCE}$$

The calculation method for $L_{Dice}$ and $L_{wCE}$ is as follows:

(10) $$L_{Dice}=1-\frac{2\sum_{i}^{N}y_{i}{\hat{y}}_{i}}{\sum_{i}^{N}{y_{i}}^{2}+\sum_{i}^{N}{{\hat{y}}_{i}}^{2}}$$

(11) $$L_{wCE}=-\sum_{i}^{N}\left[wy_{i}\cdot \log\left({\hat{y}}_{i}\right)+(1-w)(1-y_{i})\cdot \log(1-{\hat{y}}_{i})\right]$$

where $y_{i}$ represents the real label and ${\hat{y}}_{i}$ represents the posterior possibility of each individual pixel within a ground-truth or prediction map that has a total number of *N* pixels. w denotes the weight, which directly affects the final result.

Since the model is updating during the training process, using a constant weight is not suitable. In the early learning process, the model is far from accurate and with a steep initial decline in the loss function, so too large w may increase the false detection rate in the crack area. When the decline of the value of the loss function slows down, the greater weight will further improve the problem of category imbalance and increase the recall rate of crack areas. Based on this intuition, a dynamic weight updating mechanism is proposed, as detailed in Algorithm 1.
**Algorithm 1.** Dynamic cross-entropy weight updating.1. **Input**: The maximum numbers of iterations $N_{max}$ and epochs $N_{epoch}$; update interval $N_{\mathrm{interval}}=\alpha \cdot N_{\max}/N_{epoch}$; mean loss in one interval $L_{mean}$, initialized by the last $N_{\mathrm{interval}}$ iterations; the number of loss decreases $M_{L\_down}$; the number of loss increases $M_{L\_up}$; initial values are 0. **Output**: The updated weight parameters w 2. **For** $n_{iter}\;<\;N_{\max}$ **do** 3. Compute $L_{n}$ according to Equation (9) 4. **if** $\left(L_{n}<L_{mean}\right)\;$// 5. $M_{L\_down}=M_{L\_down}+1$; // the number of loss decreases 6. **else if**
$\left(L_{n}>L_{mean}\right)$ 7. $M_{L\_up}=M_{L\_up}+1$;    // the number of loss increases 8. **End if** 9. **if** $\left(M_{L\_up}+M_{L\_up}=\;=\;N_{\mathrm{interval}}\right)$ // the number of iterations meets the interval. 10. **if**
$\left(M_{L\_down}-M_{L\_up}>Thr\right)\;$// 11. $w=w+(1-\;\frac{N_{iter}}{N_{\max}})$ // increase weight 12. **else**
$w=w-(1-\;\frac{n_{iter}}{N_{\max}})$ // decrease weight 13. **end if** 14. $M_{L\_down}=0,M_{L\_up}=0$; update $L_{mean}$; 15. **end if** 16. $n_{iter}\;=\;n_{iter}\;+1$ 17. **End for**

The underlying idea behind weight adjustment is to assess, within an interval, whether the model’s loss after each iteration, compared to the average loss from the previous cycle, has increased or decreased. If the number of decreases exceeds the number of increases, the weight assigned to the crack category is further increased; conversely, it is decreased. For the robustness of the method, the weight is only increased when the difference between the number of decreases and increases exceeds a certain threshold Thr. Practically, the weight is updated at set intervals for robustness. The initial weight value $w_{initial}$ can be selected based on personal experience, or foreground–background ratio, etc.

## 4. Experimental Results and Discussion

The proposed CrackNet was evaluated on three public crack datasets, including DeepCrack [11], Crack500 [12], and CFD [43]. Moreover, we also conduct comprehensive ablation analysis on the effect of the proposed model and the dynamic weight updating mechanism based on the CFD dataset.

### 4.1. Datasets and Preprocessing

(1) DeepCrack: The dataset is composed of 537 RGB color images with manually annotated segmentations, which are of a fixed size of 544 × 384 pixels. The dataset was divided into a training set with 300 images and a testing set with 237 images.
(2) Crack500: This dataset contains 500 images and corresponding pixel-level annotations of size around 2000 × 1500 pixels, all taken by mobile phone. The dataset is divided into 250 images of training data, 50 images of validation data, and 200 images for testing.
(3) CFD: This dataset consists of 118 images of size 480 × 320, which was taken by an iPhone 5 on an urban road surface in Beijing, China. All images were annotated at pixel level. This dataset is very challenging due to the complex background, including shadows, oil spots, water stains, and lane lines. We divide 60% and 40% of this dataset into training and testing, respectively.

### 4.2. Data Augmentation

To increase the diversity of the data, we argument the datasets by random clipping, flipping, and rotation operations. Hence, the DeepCrack and Crack500 datasets were augmented by 4 times, while the CFD dataset was augmented by 2 times. Furthermore, Bilateral filter and Gaussian filter are adopted to eliminate noise interference. Due to the large image size and limited memory of GPU, each image is resized to 224 × 224 pixels. It is worth noting that the proportion of crack pixels in each database is less than 1%, which helps to examine the efficiency of the proposed approach for imbalance class segmentation.

### 4.3. Parameters

All the experiments mentioned in this paper were based on the Ubuntu 16.04.6 LTS system in NVIDIA Tesla P100 GPU with 16 G memory. Moreover, to control variables, each experiment was set to 60 epochs, a learning rate of 0.01, and the same learning rate decay strategy.

### 4.4. Performance Metrics

In experiments, the F1-score, the intersection over union (*IoU*), precision (*Pr*), recall (*Re*), and accuracy (*Acc*) are selected to evaluate the performance of the model.

(12) $$Pr=\frac{TP}{TP+FP}$$

(13) $$Re=\frac{TP}{TP+FN}$$

(14) $$F1=\frac{2\times Pr\times Re}{Pr+Re}$$

(15) $$IoU=\frac{TP}{TP+FP+FN}$$

(16) $$Acc=\frac{TP+TN}{TP+TN+FP+FN}$$

where *TP*, *FP*, and *FN* refer to the true positive, false positive, and false negative, respectively.

### 4.5. Results Comparisons

To illustrate the performance of the proposed model, we select DeepCrack [11], UNet [15], TransUNet [32], and Swin-Unet [33] to compare with ours on three public datasets. All experiments were conducted using open-source implementation, based on the principle of controlling variables, and all model parameter settings are kept consistent.

(1) Performance on the DeepCrack Dataset

The results on the DeepCrack Dataset are listed in Table 1. Obviously, the proposed CrackNet achieves the best results in four metrics, including F1, IoU, Re, and ACC. Although higher Pr could be achieved by TransUNet, our model obtained better tradeoff between precision and recall.

(2) Performance on the Crack500 Dataset:

The results on the Crack500 Dataset are listed in Table 2. Due to the high complexity of this dataset, our proposed model only has a 1% improvement in F1, but it has a significant effect on recall which it turns out solves the category imbalance to some extent.

(3) Performance on the CFD Dataset:

On the small-scale dataset CFD, the model we proposed still performs well thanks to data argumentation and the combination with CNN and transformer. Therefore, complementary integration of CNN and transformer is feasible. As can be seen from Table 3, the comparison models have significant shortcomings in recall due to category imbalance. In contrast, the CrackNet network proposed in this work shows a noticeable improvement in recall rate. From an application perspective, this would eliminate crack threats as soon as possible.

### 4.6. Qualitative Results Comparisons

To provide a more intuitive comparison of the segmentation results, this subsection visualizes the segmentation results of some sample images.

(1) Qualitative results on the DeepCrack

**Figure 6:**
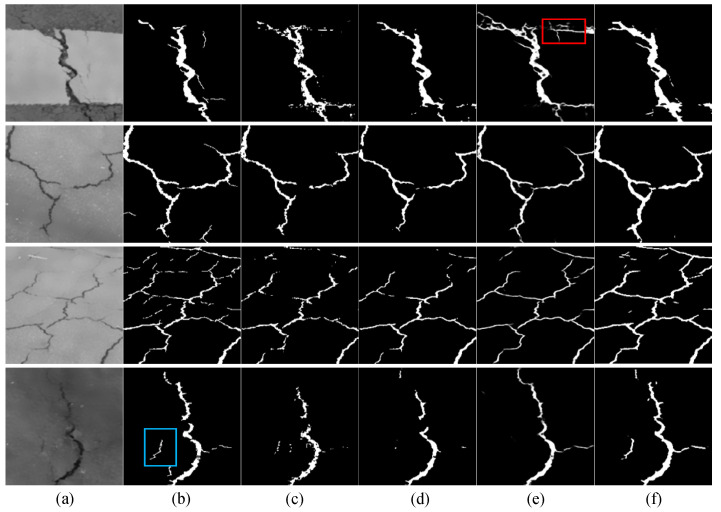
Visualization of testing results on the DeepCrack. (**a**) original image, (**b**) groundtruth, (**c**) Swin-Unet, (**d**) TransUNet, (**e**) DeepCrack, (**f**) CrackNet. The red box denotes the areas that are erroneously identified as cracks. The blue box represents the fine cracks that are prone to being overlooked.

As shown in Figure 6, it can be seen that the proposed model is particularly sensitive to narrow and elongated cracks, especificallythose located in cluttered backgrounds. For instance, in the first-row sample, where the background includes pedestrian crosswalk markings, other models (especially the DeepCrack) erroneously identify the ground markings as cracks (red box). In the fourth image, the contrast and brightness of the image are relatively low, which poses a challenge for crack segmentation. However, the proposed model still segments the cracks relatively completely and captures small cracks that other models often ignore (blue box).

(2) Qualitative results on the Crack500

**Figure 7:**
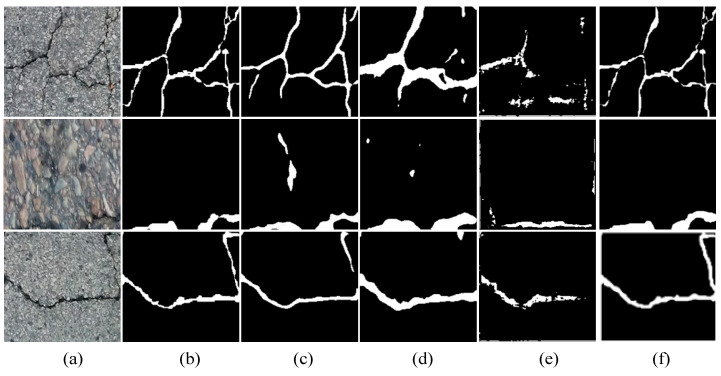
Visualization of testing results on the Crack500. (**a**) original image, (**b**) groundtruth, (**c**) Swin-Unet, (**d**) TransUNet, (**e**) DeepCrack, (**f**) CrackNet.

To better demonstrate the advantages of the model proposed in this paper, we further selected experimental results from the Crack500 dataset, as shown in Figure 7. From the figure, it can be observed that the proposed model outperforms other models in terms of its resistance to interference from noisy textured backgrounds and its ability to accurately extract narrow cracks.

(3) Qualitative results on the CFD

**Figure 8:**
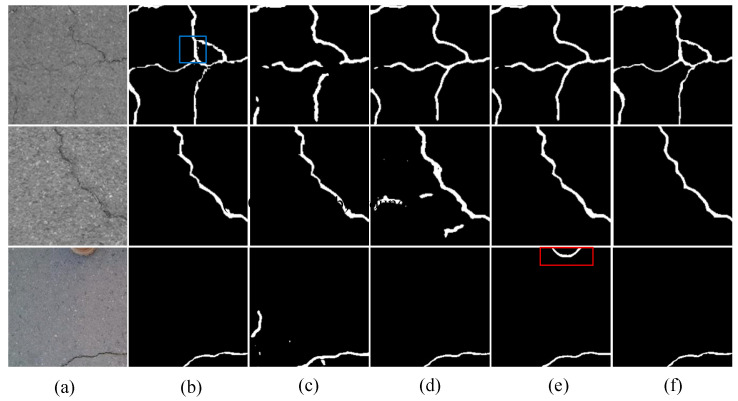
Visualization of testing results on the CFD. (**a**) original image, (**b**) groundtruth, (**c**) Swin-Unet, (**d**) TransUNet, (**e**) DeepCrack, (**f**) CrackNet. The red box denotes the areas that are erroneously identified as cracks. The blue box represents the fine cracks that are prone to being overlooked.

The experimental results on the CFD dataset are shown in Figure 8. From the figure, it is easy to observe that the proposed model demonstrates excellent detail extraction and noise resistance capabilities. For instance, in the first sample, the low contrast of the image significantly interferes with crack segmentation, but the proposed CrackNet successfully captures details that other methods overlook (highlighted in the red box). In the third sample, an error during image capture resulted in the photographer’s shoe being mistakenly included in the frame. Notably, the DeepCrack model, lacking long-distance modeling capabilities, incorrectly identifies the shoe as a crack (highlighted in the blue box).

### 4.7. Comparisons with Other Methods

The proposed method was also compared with other methods across two public datasets. The data for other methods were obtained from the best score of the original papers. The scale of the CFD dataset is very small and the crack regions in this dataset are extremely slender, posing a challenge to the model’s sensitivity in detecting fine-grained crack pixels. As shown in Table 4, our proposed CrackNet method achieved the best performance in terms of recall (Re) and F1 score, with a slightly lower precision rate (Pr) compared to other methods.

In the DeepCrack dataset, crack regions only account for a small number of pixels, and the problem of category imbalance is particularly prominent. It can be seen from Table 5 that the overall performance of the proposed network is comparable with recent specialized crack segmentation algorithms and other segmentation algorithms, and is also superior to some other segmentation algorithms.

### 4.8. Ablation Experiments

In order to evaluate the validity of each module in the proposed model, an ablation study is performed on the CFD dataset. The experimental results are shown in Table 6. From the table, it can be seen that strip pooling has a positive effect on the model’s segmentation performance for small cracks. Due to the characteristics of small cracks, the contribution to the improvement in accuracy may not be significant, but it cannot be ignored. And the proposed dynamic loss function greatly improves the problem of class imbalance, which is reflected in a significant increase in recall rate.

## 5. Conclusions

The proposed CrackNet network combines the advantages of CNN and transformer, and introduces strip pooling to better capture the slender characteristics of cracks. More importantly, to address the issue of class imbalance, we have developed a dynamic loss function during the training process. Experiments on three public datasets demonstrate the superiority of the proposed method. In future work, we plan to further test our approach on actual crack images and consider deploying it on edge devices.

However, there are still some problems to be solved in the field, such as how to effectively use the edge information. Moreover, compared to other natural images, crack images exhibit high intra-class similarity and typically involve small dataset sizes, making them prone to overfitting with excessive convolutional layers. Therefore, using pruning techniques, such as reducing the number of convolutional layers, can not only simplify the structure but also potentially enhance performance. We will consider reducing the number of convolutional layers in our future research to make the model more feasible for practical and real-time industrial applications.

## Figures and Tables

**Table 1:** Performance on the DeepCrack Dataset.

Model	F1	IoU	Pr	Re	Acc
DeepCrack [11]	0.7473	0.6132	0.6342	0.9604	0.9737
U-Net [15]	0.7887	0.6832	0.8852	0.7504	0.9837
TransUNet [32]	0.8338	0.7243	0.9034	0.7902	0.9865
Swin-Unet [33]	0.7789	0.6513	0.8691	0.7328	0.9819
CrackNet	0.8425	0.7406	0.8690	0.8424	0.9872

**Table 2:** Performance on the Crack500.

Model	F1	IoU	Pr	Re	Acc
DeepCrack [11]	0.4911	0.3425	0.3607	0.8982	0.9254
U-Net [15]	0.6693	0.5279	0.6998	0.6876	0.9672
TransUNet [32]	0.6520	0.5053	0.7014	0.6587	0.9577
Swin-Unet [33]	0.6426	0.4971	0.6915	0.6519	0.9529
CrackNet	0.6744	0.5293	0.6474	0.7595	0.9611

**Table 3:** Performance on the CFD.

Model	F1	IoU	Pr	Re	Acc
DeepCrack [11]	0.6125	0.4536	0.5151	0.8174	0.9782
U-Net [15]	0.6723	0.5182	0.6257	0.7519	0.9392
TransUNet [32]	0.6791	0.5243	0.6639	0.7122	0.9484
Swin-Unet [33]	0.6297	0.4690	0.6344	0.6574	0.9416
CrackNet	0.7035	0.5509	0.6381	0.7957	0.9502

**Table 4:** Comparison of results between CrackNet and other methods on CFD.

Methods	Pr	Re	F1
RIND [44]	0.6456	0.7534	0.6954
UCTNet [34]	0.6701	0.7205	0.6944
Crackmer [38]	0.6370	0.7237	0.6776
CrackNet	0.6381	0.7957	0.7035

**Table 5:** Comparison of results between CrackNet and other methods on DeepCrack.

Methods	Pr	Re	F1
RIND [44]	0.7896	0.8920	0.8377
EfficientNet [45]	0.6925	0.8614	0.7678
UCTNet [34]	0.8217	0.8857	0.8525
Crackmer [38]	0.8931	0.8233	0.8568
CrackNet	0.8690	0.8424	0.8425

**Table 6:** Results of the ablation experiments on the CFD.

Method			F1	IoU	Pr	Re
Baseline	SP	DWL				
√			0.6704	0.5177	0.7130	0.6765
√	√		0.6782	0.5245	0.7080	0.6893
√	√	√	0.7035	0.5509	0.6381	0.7957

## Data Availability

We use three publicly available image datasets to evaluate the detection network proposed in this paper, namely, the CFD dataset, the Crack-2 dataset, and the Crack500 dataset. They can be downloaded from the URL https://paperswithcode.com/dataset/cfd (accessed on 30 June 2024), https://universe.roboflow.com/university-bswxt/crack-bphdr?ref=ultralytics (accessed on 5 February 2024) and https://paperswithcode.com/dataset/crack500 (accessed on 30 March 2024).

## References

[1] Mohan A., Poobal S. (2018). Crack detection using image processing: A critical review and analysis. Alex. Eng. J..

[2] König J., Jenkins M., Mannion M., Barrie P., Morison G. (2022). What’s Cracking? A Review and Analysis of Deep Learning Methods for Structural Crack Segmentation, Detection and Quantification. arXiv.

[3] Pan Y., Zhang X., Cervone G., Yang L. (2018). Detection of asphalt pavement potholes and cracks based on the unmanned aerial vehicle multispectral imagery. IEEE J. Sel. Top. Appl. Earth Obs. Remote Sens..

[4] Dung C.V., Anh L.D. (2019). Autonomous concrete crack detection using deep fully convolutional neural network. Autom. Constr..

[5] Xinghao C., Wang G., Guo H., Zhang C., Wang H., Zhang L. (2019). Mfa-net: Motion feature augmented network for dynamic hand gesture recognition from skeletal data. Sensors.

[6] Wooram C., Cha Y.-J. (2019). SDDNet: Real-time crack segmentation. IEEE Trans. Ind. Electron..

[7] Cui X., Wang Q., Dai J., Xue Y., Duan Y. (2021). Intelligent crack detection based on attention mechanism in convolution neural network. Adv. Struct. Eng..

[8] Girshick R. (2015). Fast r-cnn. arXiv.

[9] Redmon J. You only look once: Unified, real-time object detection. Proceedings of the IEEE Conference on Computer Vision and Pattern Recognition.

[10] Huyan J., Li W., Tighe S., Xu Z., Zhai J. (2020). CrackU-net: A novel deep convolutional neural network for pixelwise pavement crack detection. Struct. Control Health Monit..

[11] Liu Y., Yao J., Lu X., Xie R., Li L. (2019). DeepCrack: A deep hierarchical feature learning architecture for crack segmentation. Neurocomputing.

[12] Yang F., Zhang L., Yu S., Prokhorov D., Mei X., Ling H. (2019). Feature pyramid and hierarchical boosting network for pavement crack detection. IEEE Trans. Intell. Transp. Syst..

[13] Schütt K.T., Sauceda H.E., Kindermans P.J., Tkatchenko A., Müller K.R. (2018). Schnet–a deep learning architecture for molecules and materials. J. Chem. Phys..

[14] Vijay B., Kendall A., Cipolla R. (2017). Segnet: A deep convolutional encoder-decoder architecture for image segmentation. IEEE Trans. Pattern Anal. Mach. Intell..

[15] Olaf R., Fischer P., Brox T. (2015). U-net: Convolutional networks for biomedical image segmentation. Medical Image Computing and Computer-Assisted Intervention, Proceedings of the MICCAI 2015: 18th International Conference, Part III 18, Munich, Germany, 5–9 October 2015.

[16] Wang L., Ye Y. Computer vision-based road crack detection using an improved I-UNet convolutional networks. Proceedings of the 2020 Chinese Control And Decision Conference (CCDC).

[17] Fangyu L., Wang L. (2022). UNet-based model for crack detection integrating visual explanations. Constr. Build. Mater..

[18] Fan L., Zhao H., Li Y., Li S., Zhou R., Chu W. (2022). RAO-UNet: A residual attention and octave UNet for road crack detection via balance loss. IET Intell. Transp. Syst..

[19] Lau S.L., Chong E.K., Yang X., Wang X. (2020). Automated pavement crack segmentation using u-net-based convolutional neural network. IEEE Access.

[20] Chen T., Cai Z., Zhao X., Chen C., Liang X., Zou T., Wang P. (2020). Pavement crack detection and recognition using the architecture of segNet. J. Ind. Inf. Integr..

[21] Shengyuan L., Zhao X., Zhou G. (2019). Automatic pixel-level multiple damage detection of concrete structure using fully convolutional network. Comput. Aided Civ. Infrastruct. Eng..

[22] Kang D., Benipal S.S., Gopal D.L., Cha Y.J. (2020). Hybrid pixel-level concrete crack segmentation and quantification across complex backgrounds using deep learning. Autom. Constr..

[23] Honghu C., Wang W., Deng L. (2022). Tiny-Crack-Net: A multiscale feature fusion network with attention mechanisms for segmentation of tiny cracks. Comput. Aided Civ. Infrastruct. Eng..

[24] Ashish V., Shazeer N., Parmar N., Uszkoreit J., Jones L., Gomez A.N., Kaiser Ł., Polosukhin I., Guyon I., Von Luxburg U., Bengio S., Wallach H., Fergus R., Vishwanathan S., Garnett R. (2017). Attention is all you need. Advances in Neural Information Processing Systems.

[25] Dosovitskiy A., Beyer L., Kolesnikov A., Weissenborn D., Zhai X., Unterthiner T., Dehghani M., Minderer M., Heigold G., Gelly S. (2020). An image is worth 16x16 words: Transformers for image recognition at scale. arXiv.

[26] Liu Z., Lin Y., Cao Y., Hu H., Wei Y., Zhang Z., Lin S., Guo B. Swin transformer: Hierarchical vision transformer using shifted windows. Proceedings of the IEEE/CVF International Conference on Computer Vision.

[27] Nikita K., Kaiser Ł., Levskaya A. (2020). Reformer: The efficient transformer. arXiv.

[28] Wenjun W., Su C. (2022). Automatic concrete crack segmentation model based on transformer. Autom. Constr..

[29] Shamsabadi E.A., Xu C., Rao A.S., Nguyen T., Ngo T., Dias-da-Costa D. (2022). Vision transformer-based autonomous crack detection on asphalt and concrete surfaces. Autom. Constr..

[30] Dang L.M., Wang H., Li Y., Nguyen T.N., Moon H. (2022). DefectTR: End-to-end defect detection for sewage networks using a transformer. Constr. Build. Mater..

[31] Qi H., Kong X., Jin Z., Zhang J., Wang Z. A Vision-Transformer-Based Convex Variational Network for Bridge Pavement Defect Segmentation. Proceedings of the IEEE Transactions on Intelligent Transportation Systems.

[32] Chen J., Lu Y., Yu Q., Luo X., Adeli E., Wang Y., Lu L., Yuille A.L., Zhou Y. (2021). Transunet: Transformers make strong encoders for medical image segmentation. arXiv.

[33] Cao H., Wang Y., Chen J., Jiang D., Zhang X., Tian Q., Wang M. (2023). Swin-unet: Unet-like pure transformer for medical image segmentation. Computer Vision, Proceedings of the ECCV 2022 Workshops, Part III, Tel Aviv, Israel, 23–27 October 2022.

[34] Guo X., Lin X., Yang X., Yu L., Cheng K.-T., Yan Z. (2024). UCTNet: Uncertainty-guided CNN-Transformer hybrid networks for medical image segmentation. Pattern Recognit..

[35] Wu M., Jia M., Wang J. (2022). TMCrack-Net: A U-shaped network with a feature pyramid and transformer for mural crack segmentation. Appl. Sci..

[36] Xiang C., Guo J., Cao R., Deng L. (2023). A crack-segmentation algorithm fusing transformers and convolutional neural networks for complex detection scenarios. Autom. Constr..

[37] Liu H., Miao X., Mertz C., Xu C., Kong H. Crackformer: Transformer network for fine-grained crack detection. Proceedings of the IEEE/CVF International Conference on Computer Vision.

[38] Wang J., Zeng Z., Sharma P.K., Alfarraj O., Tolba A., Zhang J., Wang L. (2024). Dual-path network combining CNN and transformer for pavement crack segmentation. Autom. Constr..

[39] Xie S., Tu Z. Holistically-Nested Edge Detection. Proceedings of the IEEE International Conference on Computer Vision.

[40] Jie F., Qu B., Yuan Y. (2021). Distribution equalization learning mechanism for road crack detection. Neurocomputing.

[41] Li K., Wang B., Tian Y., Qi Z. (2021). Fast and Accurate Road Crack Detection Based on Adaptive Cost-Sensitive Loss Function. IEEE Trans. Cybern..

[42] Hou Q., Zhang L., Cheng M.M., Feng J. Strip pooling: Rethinking spatial pooling for scene parsing. Proceedings of the IEEE/CVF Conference on Computer Vision and Pattern Recognition.

[43] Shi Y., Cui L., Qi Z., Meng F., Chen Z. (2016). Automatic road crack detection using random structured forests. IEEE Tran. Intel. Trans. Syst..

[44] Mengyang P., Huang Y., Guan Q., Ling H. Rindnet: Edge detection for discontinuity in reflectance, illumination, normal and depth. Proceedings of the IEEE/CVF International Conference on Computer Vision.

[45] Koonce B. (2021). EfficientNet. Convolutional Neural Networks with Swift for Tensorflow: Image Recognition and Dataset Categorization.

